# Long-term outcomes of bioabsorbable polylactide scaffold-based versus conventional arthroplasty for trapeziometacarpal osteoarthritis

**DOI:** 10.3205/iprs000196

**Published:** 2026-09-24

**Authors:** Antek Nicklas, Feras Taqatqeh, Adrian Dragu, Bilal Msallem

**Affiliations:** 1 UniversityCenter for Orthopedics, Trauma and Plastic Surgery, Faculty of Medicine and University Hospital Carl Gustav Carus, TU Dresden, Germany; 2 LipoXpert Clinic, Amman, Jordan

**Keywords:** arthroplasty, absorbable implants, joint replacement, osteoarthritis, precision medicine, RegJoint™

## Abstract

**Introduction:**

With rapid advancements in hand surgery, new approaches for treating trapeziometacarpal osteoarthritis are continuously evaluated. RegJoint™ is a bioabsorbable polylactide scaffold for scaphometacarpal interposition after trapeziectomy. However, the long-term outcomes of arthroplasty with bioabsorbable polylactide scaffolds compared to conventional arthroplasty techniques remain uncertain.

**Materials and methods:**

A comprehensive clinical and radiological evaluation was conducted on 18 patients, with a mean follow-up duration of 45.5 months. Patient-reported outcomes were assessed using the EQ-5D, DASH, and SF-36 questionnaires. The clinical assessment encompassed measurements of range of motion, opposition, first-ray length, key-pinch and grip strength.

**Results:**

The two groups showed comparable results with respect to the EQ-5D, DASH, SF-36, and clinical assessments.

**Conclusions:**

Arthroplasty with bioabsorbable polylactide scaffolds and conventional arthroplasty techniques demonstrate comparable long-term outcomes in this small cohort study, and neither approach showed a clear advantage over the other. Given these findings, no definitive recommendation can be made between the different techniques. However, the RegJoint™ approach avoids the need for a second donor site, preserves native tissue, and lowers the risk of donor-site infection, indicating a favorable tendency toward its use. Further investigation into its potential benefits, including cost-effectiveness and procedural efficiency relative to implant-based joint replacement, is warranted.

## Zusammenfassung

**Einleitung:**

Mit den rasanten Fortschritten in der Handchirurgie werden kontinuierlich neue Verfahren zur Behandlung der Rhizarthrose evaluiert. RegJoint™ ist ein bioresorbierbares Polylactid-Scaffold zur skaphometakarpalen Interposition nach Trapezektomie. Die Langzeitergebnisse dieser Arthroplastik mit bioresorbierbaren Polylactid-Scaffolds im Vergleich zu konventionellen Arthroplastikverfahren sind jedoch derzeit noch Gegenstand der Forschung.

**Material und Methoden:**

Insgesamt wurden 18 Patientinnen und Patienten über einen mittleren Nachbeobachtungszeit von 45,5 Monaten klinisch und radiologisch untersucht. mit einer mittleren Nachbeobachtungszeit von 45,5 Monaten. Die patientenspezifischen Endpunkte wurden mithilfe der Fragebögen EQ-5D, DASH und SF-36 erfasst. Die klinische Untersuchung umfasste die Messung des Bewegungsumfangs, der Oppositionsfähigkeit, der Länge des ersten Strahls sowie der Schlüsselgriff- und Handkraft.

**Ergebnisse:**

Beide Gruppen zeigten vergleichbare Ergebnisse hinsichtlich der patientenberichteten Endpunkte im EQ-5D, DASH und SF-36 sowie der klinischen Untersuchungsparameter.

**Schlussfolgerung:**

In dieser Kohortenstudie mit geringer Fallzahl erzielten die Arthroplastik mit bioresorbierbarem Polylactid-Scaffold und die konventionellen Arthroplastikverfahren vergleichbare Langzeitergebnisse. Keines der beiden Verfahren erwies sich dem anderen als eindeutig überlegen. Angesichts dieser Ergebnisse kann keine eindeutige Empfehlung für eine der verschiedenen Techniken ausgesprochen werden. Das RegJoint™-Verfahren vermeidet jedoch die Notwendigkeit einer zweiten Entnahmestelle, erhält das native Gewebe und reduziert das Risiko einer Infektion an der Entnahmestelle, was für eine tendenzielle Bevorzugung seiner Anwendung spricht. Weitere Untersuchungen zu den potenziellen Vorteilen, einschließlich der Kosteneffektivität und der Verfahrenseffizienz im Vergleich zum implantatbasierten Gelenkersatz, sind wünschenswert.

## 1 Introduction

The carpometacarpal joint of the thumb is a concave saddle joint between the first metacarpale and the trapezium, which ensures the multiplanar mobility of the thumb. Given the high strain placed on the thumb throughout its life, with gripping forces of up to 120 kg and pointed grip forces of up to 12 kg, it is unsurprising that this joint is predisposed to degeneration in the form of osteoarthritis [1]. The trapeziometacarpal osteoarthritis, also known as rhizarthrosis, is a common disease, accounting for 10% of all arthritic manifestations, and the prevalence increases with age and female gender [2], [3]. The female sex is predisposed to its development, with a significant increase in frequency with age. In women over the age of 80, the prevalence of radiologically diagnosed rhizarthrosis is 91%, and from the age of 91, the prevalence is 100% [4]. Insufficiency of the anterior oblique ligament has also been demonstrated to be associated with the progression of thumb saddle joint arthrosis [5], [6].

Patients suffering from advanced rhizarthrosis experience progressive pain in the area of the thumb saddle joint, which increases in severity with the progression of the disease, resulting in a considerable restriction of everyday life activities. In addition to the initial pain symptoms, patients may also experience a loss of strength in their pinch and grip. Clinically, the final stage of rhizarthrosis is characterized by a restriction of movement with an adduction contracture. Radiologically, rhizarthrosis is classified into 4 stages according to the Eaton and Littler classification system [7]. This classification system incorporates the presence of characteristic signs of osteoarthritis, including osteophyte formation, subchondral sclerosis, joint space narrowing, and the presence of free joint bodies depending on their size (i.e. those measuring less than or greater than 2 mm) and osteoarthritis in the scapho-trapezo-trapezoidal joint [8]. Despite the frequency of the disease and the high level of suffering experienced by patients, there is currently at the time of writing this article this article no guideline of the Association of the Scientific Medical Societies in Germany on treatment and therapy recommendations for rhizarthrosis. Consequently, a multitude of surgical procedures are at the disposal of the practitioner in the event that conservative methods have been unsuccessful. These procedures are characterized by the performance of a trapeziectomy, which can be performed with or without interposition. Several distinct methods of surgical intervention for rhizarthrosis are employed at our clinic. Following trapeziectomy, the resulting cavity can be filled with either autologous or alloplastic materials, such as the biodegradable polylactic acid-based scaffold (RegJoint™), which the manufacturer asserts can support the development of a pseudo-joint within three months. Over time, flexible connective tissue forms in the former joint cavity. Despite the development of novel surgical concepts and techniques, the space created by the resected trapezium was typically filled with autologous material. The application of a bioabsorbable scaffold obviates the need for tendon resection, thereby preventing donor site morbidity. This approach preserves hand function, distinguishing it from traditional surgical techniques. 

The resection-suspension arthroplasty of the carpometacarpal joint of the thumb, as originally described by Epping [9], offers a technique for transosseous suspension of the first metacarpal. The procedure begins with the excision of the trapezium, followed by reconstruction of the intermetacarpal ligaments I–II using the flexor carpi radialis (FCR) tendon. An alternative approach employs the palmaris longus (PL) tendon for similar reconstruction. The resection-suspension arthroplasty, popularized by Lundborg [10], has also become a widely accepted standard of care. This technique utilizes the abductor pollicis longus (APL) tendon for suspension. However, these techniques are often associated with a high rate of postoperative loss of function and strength in the affected thumb.

At our institution, patients underwent the aforementioned conventional arthroplasty techniques from 2017 to 2023, before transitioning to the RegJoint™ implantation. Long-term patient data have been collected and compared. If patient satisfaction is comparable between the surgical approaches, it is hypothesized that preserving autologous tissue may better preserve strength and functionality, or at least cause less impairment by sparing the autologous tissue. There is limited data on long-term functionality, strength, and patient satisfaction following RegJoint™ implantation. The present study was designed as a pilot study to evaluate postoperative functionality, strength, and patient satisfaction following surgical treatment for trapeziometacarpal osteoarthritis.

## 2 Material and methods

Eighteen consecutive patients with osteoarthritis of the trapeziometacarpal joint were surgically treated at our institution between 2017 and 2023 and the participants provided informed consent to participate in this study. All methods were performed in accordance with the relevant guidelines and regulations. The indication for surgery was the failure of conservative management. The surgical techniques were carried out by three experienced surgeons. All patients had radiologically confirmed Stage III or IV rhizarthrosis, as classified by Eaton and Littler. The patient cohort is detailed below (Table 1 (Tab. 1) and Table 2 (Tab. 2)).

The objective of this study is to assess postoperative patient satisfaction and evaluate functional outcomes. Patient-reported outcomes were measured using the EQ-5D, DASH, and SF-36 questionnaires in their validated German translations. A comparative analysis of patient satisfaction and functional parameters was performed. Ethical approval was obtained from the Ethics Committee at the TU Dresden. The research was conducted in accordance with the ethical standards of the Declaration of Helsinki.

### 2.1 Operative technique: Arthroplasty with bioabsorbable polylactide scaffold (RegJoint™)

Arthroplasty using a bioresorbable polylactide scaffold is a surgical approach for treating joint degeneration. The procedure begins with preoperative planning, including imaging to assess the condition of the joint. A straight radial incision is made over the first metacarpal and trapezium to access the damaged joint. After exposing the joint, the trapezium and surrounding osteophytes are removed. A biodegradable polylactic acid implant is then inserted into the joint to create a temporary scaffold, stabilizing the bones in their proper position. The scaffold is securely fixed, and the joint is closed in layers (Figure 1 (Fig. 1)). 

Postoperatively, the joint is immobilized in a splint for two weeks. Over time, the scaffold is resorbed by the body, with scarring ensuring the bones remain in position. Postoperative care includes rehabilitation and physiotherapy to facilitate recovery and restore joint function. The X-ray images of a patient at various stages of this procedure are illustrated below (Figure 2 (Fig. 2)).

### 2.2 Operative technique: Conventional arthroplasty methods

Epping resection-suspension arthroplasty is a widely used surgical technique for treating advanced osteoarthritis of the thumb's metacarpal joint, especially when conservative treatments have failed. The procedure begins with steps leading to a complete trapeziectomy. A distally pedicled slip of the FCR tendon is then harvested through a second, smaller incision made proximally at the wrist, where the tendon is identified and partially excised. Following tendon harvest, a hole is drilled obliquely in the first metacarpal to facilitate secure tendon suspension. The tendon is then inserted to reconstruct the intermetacarpal ligaments I-II, providing stability. An alternative modification of the procedure involves using the PL tendon.

Lundborg resection-suspension arthroplasty, a different technique, begins with a trapeziectomy. A 10 cm incision is made proximal to the wrist to identify the APL tendon and harvest a slip. This tendon is then attached to the metacarpal bone of the thumb and surrounding structures to stabilize the bones and maintain their alignment. The tendon helps preserve function by allowing the thumb to retain a functional range of motion while alleviating pain. Postoperative immobilization of the thumb is essential for proper healing, followed by a rehabilitation period to restore strength, mobility, and function. The X-ray images of a patient at various stages of this procedure are illustrated below (Figure 3 (Fig. 3)).

### 2.3 Data collection

Patients who had previously undergone surgical treatment for trapeziometacarpal osteoarthritis were contacted by telephone to invite their participation in this study. Upon entry, patients were assessed by independent physicians, who had not been involved in their surgery, and were interviewed regarding their personal and medical history. Participants were required to complete three questionnaires, and their clinical and functional status was evaluated.

### 2.4 Evaluation of health status and health-related quality of life

A considerable body of literature exists describing various scoring systems used to evaluate patient satisfaction and assess quality of life. In this study, the Short Form-36 (SF-36) Health Questionnaire was employed. The SF-36 is a widely recognized tool for measuring health-related quality of life and serves as a follow-up instrument for evaluating the effectiveness of individual therapies. Originally developed by the RAND Corporation as part of the Medical Outcomes Study, the SF-36 has undergone multiple adaptations, broadening its applicability [11], [12]. It comprises 36 items, organized into eight health-status subscales: physical functioning, role limitations due to physical health, bodily pain, general health perception, vitality, social functioning, role limitations due to emotional health, and mental health.

The EQ-5D, developed by the EuroQol-Group, is a standardized measure of health-related quality of life designed to provide a simple, generic questionnaire. It includes five domains: mobility, self-care, usual activities, pain/discomfort, and depression/anxiety. Country-specific algorithms based on societal preferences for health states are used to calculate utility values. The EQ-5D also includes a visual analog scale (VAS) that records the respondent’s self-rated health status on a graduated scale (0–100), ranging from the worst to the best imaginable health. The EQ-5D-5L was used in this study to determine utility values.

### 2.5 Evaluation of the function of the hand

A wide range of scoring systems for hand function has been described in the literature. This study utilizes the Disabilities of the Arm, Shoulder, and Hand (DASH) questionnaire. Developed in 1996, the DASH is a standardized tool for assessing the functional impact of musculoskeletal disorders and injuries. The initial version was created by the Council of Musculoskeletal Specialty Societies, the American Academy of Orthopaedic Surgeons, and the Institute for Work and Health Canada [13]. The DASH consists of 30 items and is widely used to evaluate a patient's ability to perform daily tasks involving the arms.

Additionally, the study incorporates the Kapandji score and grip as well as key-pinch strength measurements. For the radiological assessment, the height of the trapezial space was evaluated.

### 2.6 Statistics

Descriptive statistical analyses were conducted, summarizing the mean, standard deviation (SD), median, and range (minimum and maximum values) for both technologies. The Mann-Whitney U test was employed to assess the normality of data distribution. All analyses were performed using SPSS statistical software (version 30, SPSS Inc., Chicago, IL, USA).

## 3 Results

A summary of the relevant characteristics and statistical results of this study are presented below.

### 3.1 Results of arthroplasty with bioabsorbable polylactide scaffold (RegJoint™)

A total of seven patients (n=7) who had previously undergone the procedure successfully agreed to participate in follow-up evaluation. The mean age of this cohort was 63.4 years (SD ± 9.1). The mean operative time was 56.6 minutes (SD ± 10.1), and the mean immobilization period was 30.0 days (SD ± 5.3).

Functional outcomes were measured using the DASH questionnaire, with a mean total DASH score of 29.7 (SD ± 25.7). The DASH Work module scored an average of 22.9 (SD ± 16.6), and the DASH Sports/Performing Arts module averaged 25.0 (SD ± 30.6). 

Health-related quality of life was assessed using the EuroQol five-dimensional questionnaire (EQ-5D). The mean EQ-5D VAS-score was 81.4 (SD ± 11.4), and the EQ-5D-5L index score averaged 0.756 (SD ± 0.283).

The SF-36 health survey yielded a mean score of 55.3 (SD ± 22.7). The additional SF-36 scale values for the RegJoint™ group are presented below (Table 3 (Tab. 3)).

For radiological assessment, conventional radiography of the first ray was performed with standard anteroposterior and lateral views. The metacarpal-to-scaphoid distance on follow-up radiographs was measured, revealing a mean value of 2.8 mm. Range of motion was assessed, with thumb opposition evaluated using the Kapandji score, which yielded a mean of 9.6. Strength was measured through key-pinch and grip strength tests, with mean scores of 4.0 kg and 27.4 kg, respectively (Table 4 (Tab. 4)).

### 3.2 Results of arthroplasty with conventional methods

A total of seven patients (n=11) who had previously undergone the procedure successfully agreed to participate in follow-up evaluation. The mean age of this cohort was 67.2 years (SD ± 8.3). The mean operative time was 82.5 minutes (SD ± 22.0), and the mean immobilization period was 36.3 days (SD ± 9.3).

Functional outcomes were measured using the DASH questionnaire, with a mean total DASH score of 31.0 (SD ± 28.7). The DASH Work module scored an average of 29.7 (SD ± 34.4), and the DASH Sports/Performing Arts module averaged 31.3 (SD ± 31.9). 

Health-related quality of life was assessed using the EuroQol five-dimensional questionnaire (EQ-5D). The mean EQ-5D VAS-score was 60.3 (SD ± 29.5), and the EQ-5D-5L index score averaged 0.753 (SD ± 0.261).

The SF-36 health survey yielded a mean score of 54.2 (SD ± 15.6). The additional SF-36 scale values for the Arthroplasty with resection-suspension group are presented below (Table 5 (Tab. 5)).

The mean metacarpal-to-scaphoid distance on follow-up radiographs was 3.2 mm. Range of motion, including thumb opposition (Kapandji score), had a mean of 9.3. Strength was assessed via key-pinch and grip tests, with mean scores of 4.1 kg and 23.3 kg, respectively (Table 6 (Tab. 6)).

## 4 Discussion

The surgical treatment of hand osteoarthritis remains a critical topic in hand surgery due to the high prevalence of this condition and its significant impact on hand function and quality of life [14]. When conservative methods fail to alleviate pain and restore function, surgery becomes inevitable. Among the many surgical approaches available, RegJoint™ implantation and resection-suspension arthroplasty represents two distinctly different but widely used options. While both share the ultimate goal of relieving pain, preserving motion, and improving hand function, their methods, and materials differ in key ways [10], [15].

RegJoint™ treatment is an example of a modern, implant-based strategy. It involves the insertion of a bioresorbable spacer made from highly porous poly-L/D-lactide into the joint space following trapeziectomy. The implant serves as a temporary structure to maintain joint height, reduce mechanical impingement, and promote fibrous tissue growth, ultimately forming a functional pseudoarthrosis. The key advantages of this technique include its relatively minimally invasive nature, shorter operative time, and the avoidance of donor site morbidity since no autologous tissue is harvested. These benefits can translate into faster recovery times and reduced postoperative discomfort for the patient. Moreover, RegJoint™ is particularly a compelling option for surgeons who prefer standardized implant-based techniques. A pilot study from 2020 comparing percutaneous stabilization using Herbert screws with RegJoint™ implants demonstrated that the latter was associated with less pain, improved limb function, and higher grip strength [16]. However, as a relatively recent innovation, long-term clinical outcome data are limited, especially in terms of implant durability, late complications, and comparison with traditional techniques. However, the RegJoint™ procedure has been reported to be associated with adverse tissue reactions following trapeziectomy [17]. Although clinical outcomes with the RegJoint™ procedure are often reported as satisfactory, the potential risk of foreign-body reactions and osteolysis warrants attention [18]. Contrary to other reports, no adverse soft tissue reactions or significant bone erosion were observed [19]. Overall, there seem to be a small risk of foreign body reaction, infection, or implant migration. Thus, patient selection and surgeon experience remain crucial for achieving optimal outcomes.

In contrast, resection-suspension arthroplasty remains a time-tested surgical approach that has been widely adopted due to its consistency and strong long-term results. The procedure involves a trapeziectomy, followed by the use of a slip from a tendon to suspend and support the base of the first metacarpal. This creates a sling that stabilizes the thumb and prevents proximal migration, while also serving as a biological interposition to prevent painful bone-on-bone contact. Because no synthetic materials are used, the procedure avoids implant-related complications, and it is considered a biologically robust and durable solution. Clinical studies have shown that Lundborg arthroplasty can offer excellent pain relief, preservation of thumb function, and strong grip strength in the long term [20], [21]. However, this technique is technically more demanding, requiring precise tendon harvesting, tunneling, and secure fixation. Additionally, because of tendon involvement, there is a risk of donor site morbidity, and rehabilitation may be longer and more intensive than with implant-based procedures [20]. In patients with tendon pathology, inflammatory arthritis, or limited healing capacity, the resection-suspension arthroplasty may be less suitable. It should be noted that implant-based surgical therapies incur higher costs than the conventional techniques. However, the resection-suspension arthroplasty may represent a more compelling option in settings with limited resources.

This study provides a comprehensive and long-term follow-up analysis of several arthroplasty techniques. No major differences were identified between the groups, except for a shorter mean operative time with the RegJoint™ procedure (56.6 minutes) than with the conventional techniques (82.5 minutes). However, because different surgeons performed these procedures, this finding should be interpreted with caution. Apart from operative time, no notable differences were observed. Both groups yielded comparable results in EQ-5D, DASH, and SF-36 scores, as well as in clinical assessments including range of motion, opposition, first-ray length, key-pinch, and grip strength.

A recent 15-year follow-up randomized controlled trial comparing RegJoint™ and tendon interposition also found comparable long-term outcomes between the two groups, with no significant differences [22]. Another retrospective study compared first metacarpal suspension arthroplasty with RegJoint™ interposition in 34 patients, with a mean follow-up of 5.3 years, revealing no significant differences in patient-reported outcomes, clinical assessments, or radiographic findings between the two groups, except for a significant improvement in palmar abduction in the RegJoint™ group [23]. A retrospective study aimed to assess the clinical and radiological outcomes of trapeziectomy with RegJoint™ interposition, demonstrating pain relief and favorable functional results in most patients, as indicated by the Quick DASH scale [24]. All of which are consistent with the results of the present long-term comparative study.

Despite these differences, each technique has a valuable place in the modern surgical management of rhizarthrosis. No single approach is ideal for all patients, and the decision-making process should be comprehensive, individualized, and evidence-based. In clinical practice, RegJoint™ may be preferred in patients seeking less invasive options, particularly those with moderate disease or low functional demands. Its simplified surgical technique and shorter recovery make it attractive in day-surgery settings.

The findings of this long-term follow-up study are subject to several limitations. A primary limitation is the small sample size, which constrains the statistical power and generalizability of the results. Institutional records initially identified over 50 patients. However, following telephone contact, some individuals declined participation primarily due to travel-related inconveniences for follow-up appointments, while others, unfortunately, had passed away. Also, the involvement of three different surgeons in performing the procedures may have contributed to variations in the results.

Advancements in biomaterials and surgical methodologies have the potential to further refine implant-based treatments. Additionally, the evolution of minimally invasive tendon suspension techniques may lead to a reduction in donor site morbidity. The Touch^®^ dual-mobility trapeziometacarpal prosthesis has been demonstrated to engender significant and sustained improvements in pain relief, hand function, patient satisfaction and the occurrence of complications [25]. Given the rapid advancement of high-tech innovations including point-of-care 3D printing, robotics or artificial intelligence, standardized implant-based techniques are likely to gain further prominence and may become the preferred approach in the future. Already robot-guided systems achieve significantly higher accuracy in osteotomies than conventional cutting-guides and could gain attraction in hand surgery [26]. The advantages of standardized implant-based techniques in modern medicine include improved consistency, enhanced predictability of outcomes, reduced variability in surgical results, and streamlined training and implementation, making them often the preferred choice in many clinical settings. 

## 5 Conclusion

It is evident that both RegJoint™ implantation and conventional arthroplasty are efficacious techniques when appropriately indicated and performed by skilled practitioners. Within this small cohort, long-term outcomes were comparable across groups, and no major differences were detected. Each technique has its own strengths and limitations. Given these findings, no definitive recommendation can be made between the two groups. 

Rather than perceiving them as rivals, they should be regarded as supplementary options within a more extensive surgical repertoire. However, as minimally invasive methods are increasingly utilized in surgical procedures and inpatient treatments are increasingly performed in day-surgery settings, there may be a tendency towards implant-based interventions.

## Abbreviations

- APL: Abductor Pollicis Longus
- DASH: Disabilities of Arm, Shoulder and Hand
- EQ-5D: EuroQol-5D
- FCR: Flexor Carpi Radialis
- PL: Palmaris Longus
- SF-36: Short Form-36
- SD: Standard deviation
- RAND: Research AND Development
- VAS: Visual Analogue Scale

## Notes

### Competing interests

The authors declare that they have no competing interests.

### Author contributions

AN: Conceptualization, data curation, formal analysis, investigation, methodology, project administration, resources, software, supervision, validation, visualization, writing – review and editing. FT: Writing – review and editing. AD: Writing – review and editing. BM: Conceptualization, data curation, formal analysis, funding acquisition, investigation, methodology, project administration, resources, software, supervision, validation, visualization, writing – original draft, writing – review and editing.

### Funding

The Article Processing Charge was funded by the joint publication funds of the TU Dresden, including Carl Gustav Carus Faculty of Medicine, and the SLUB Dresden as well as the Open Access Publication Funding of the DFG.

### Data availability

The original contributions presented in the study are included in the article, further inquiries can be directed to the corresponding author.

## Figures and Tables

**Table 1:**
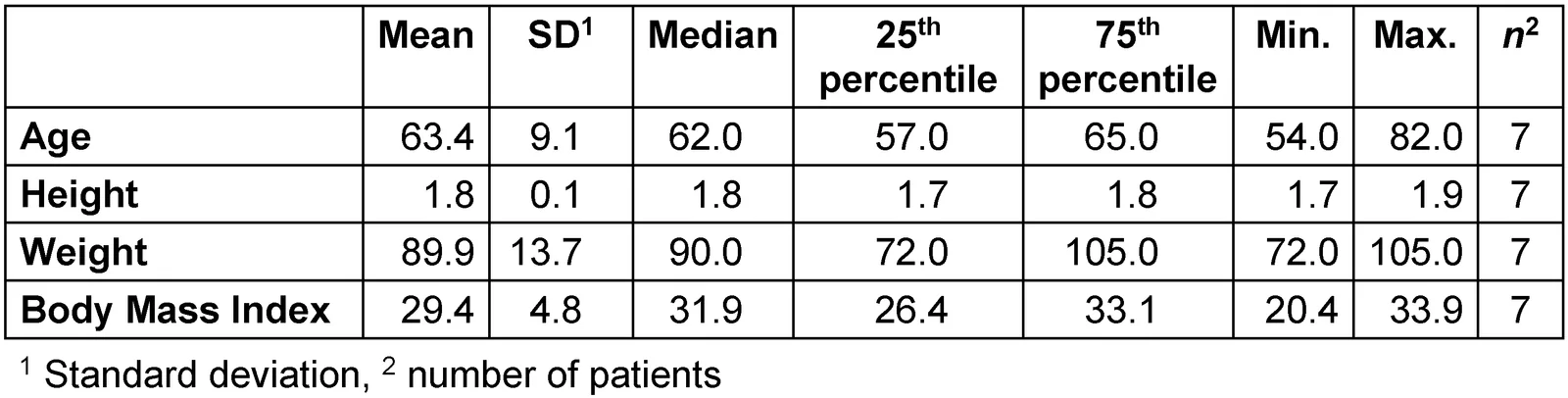
Patient cohort of the RegJoint™ group

**Table 2:**
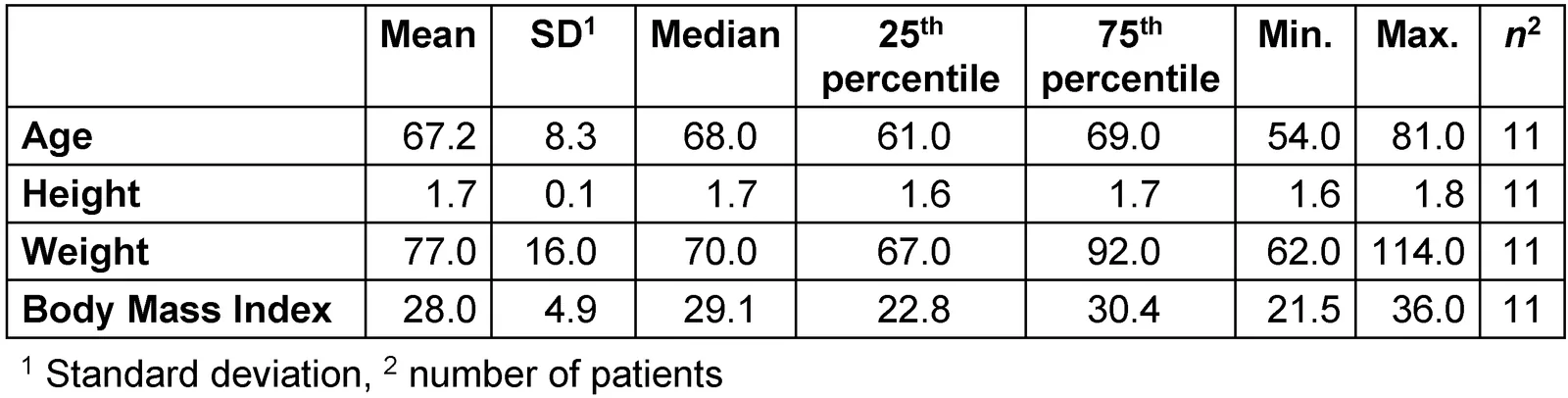
Patient cohort of the conventional group

**Table 3:**
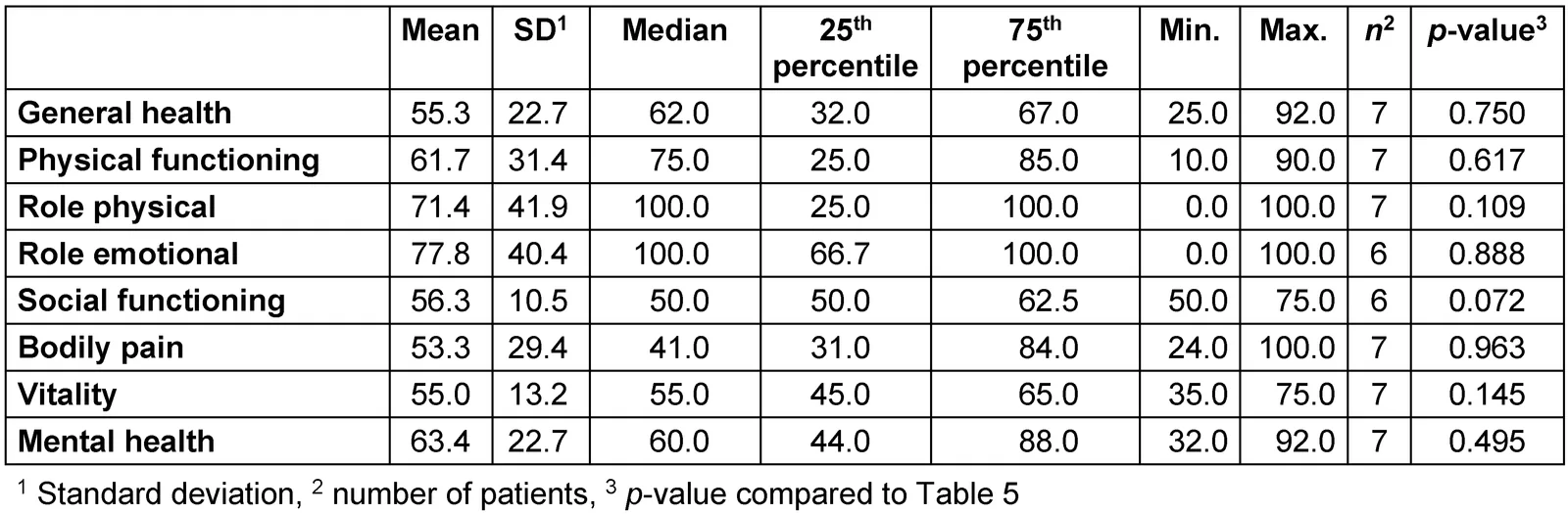
Values of SF-36 scales for the RegJoint™ group

**Table 4:**
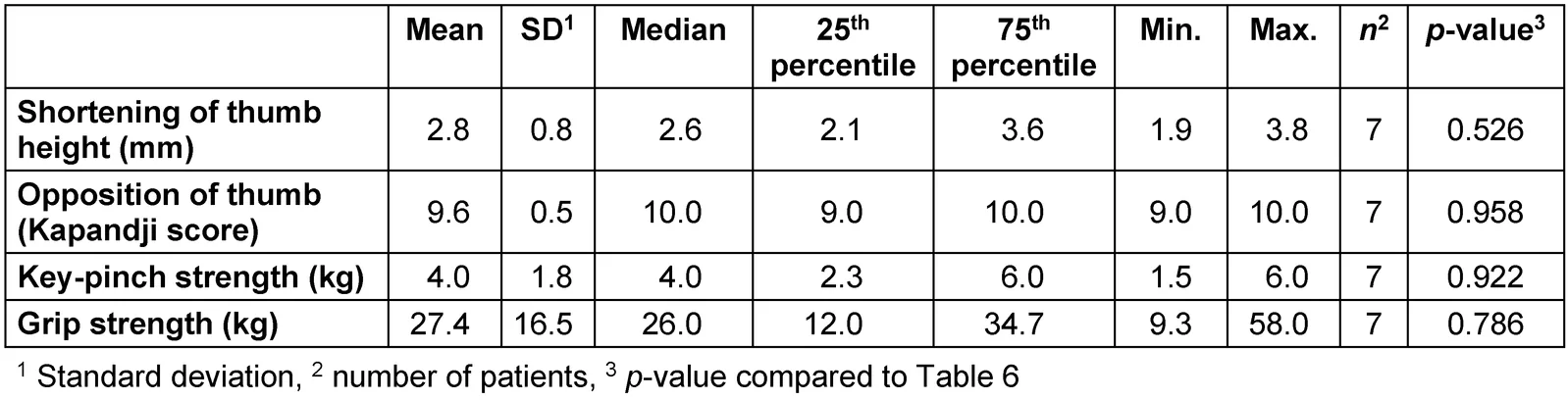
Clinical assessment for the RegJoint™ group

**Table 5:**
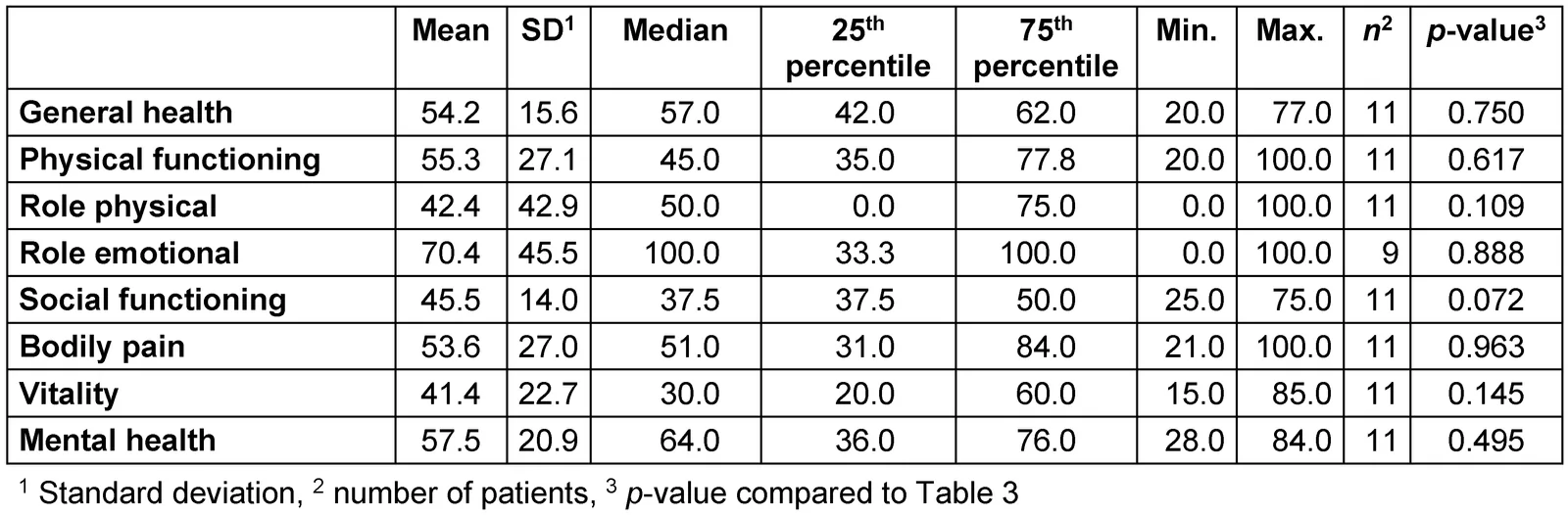
Values of SF-36 scales for the conventional group

**Table 6:**
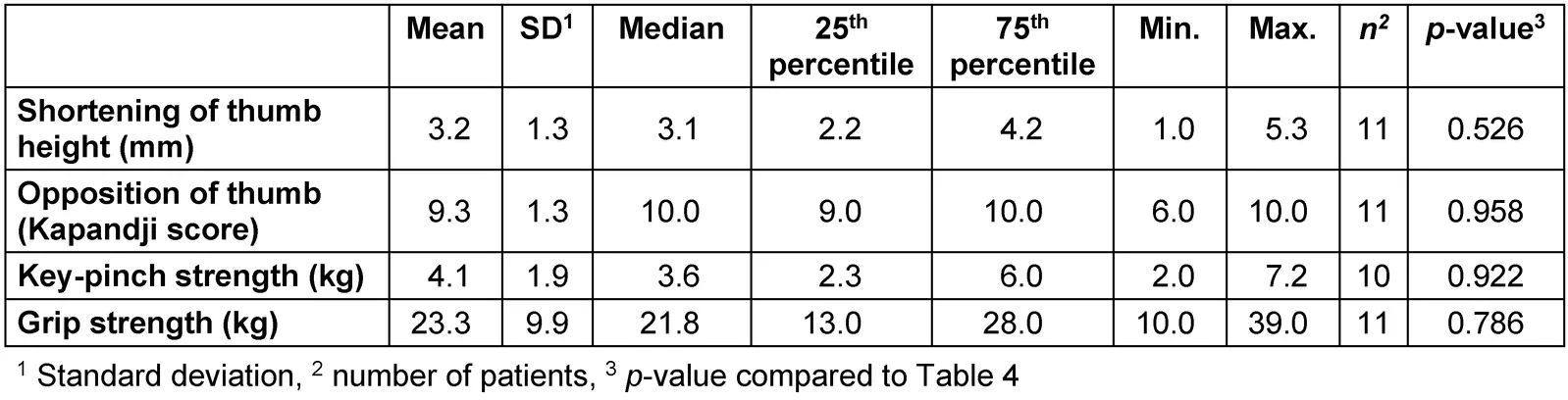
Clinical assessment for the conventional group

**Figure 1:**
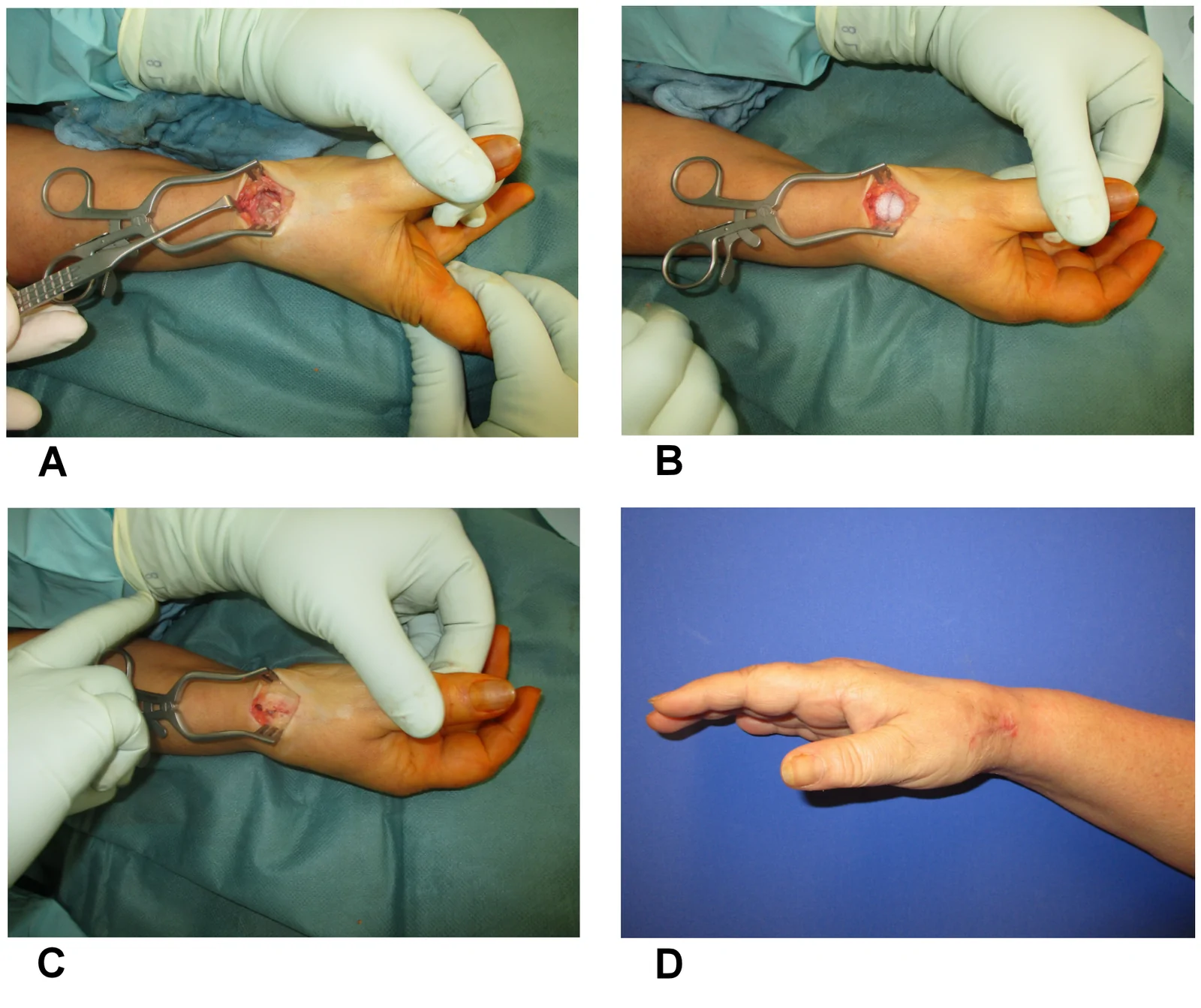
Intraoperative images following trapeziectomy (A); insertion of bioabsorbable polylactide scaffolds (RegJoint™) (B); wound closure (C); scar appearance one-month post-surgery (D).

**Figure 2:**
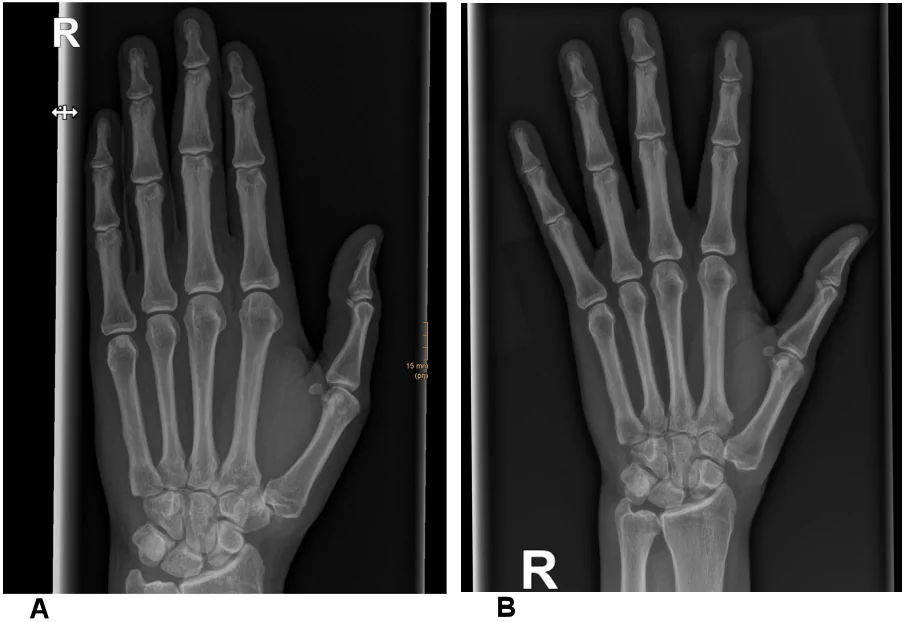
Preoperative X-ray showing joint destruction at Eaton stage III (A); postoperative X-ray following arthroplasty with bioabsorbable polylactide scaffold (RegJoint™) taken two years post-surgery (B).

**Figure 3:**
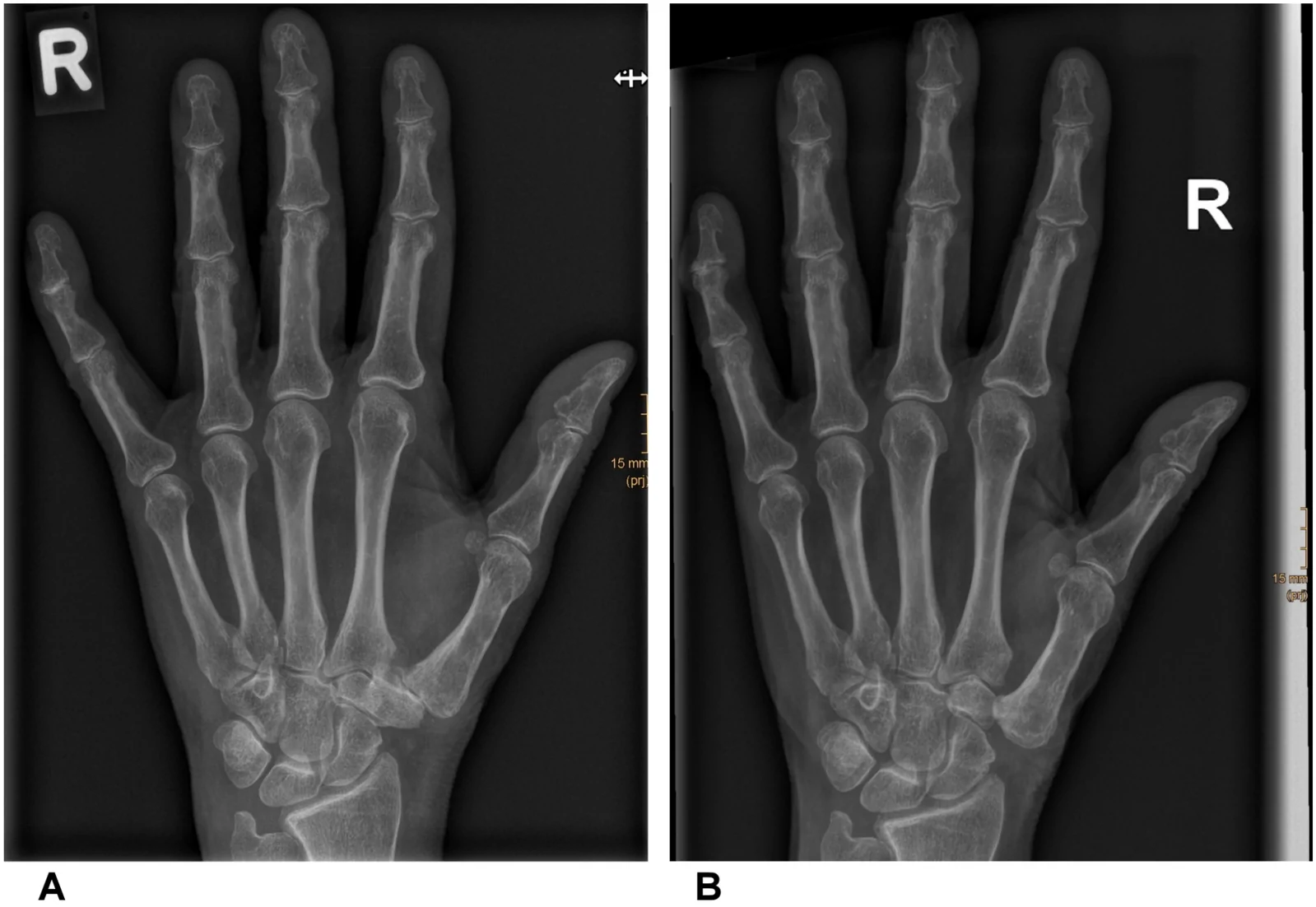
Preoperative X-ray showing joint destruction at Eaton stage IV (A); postoperative X-ray following arthroplasty with Lundborg resection-suspension taken two years post-surgery (B).

## References

[1] Cooney WP, Chao EY (1977). Biomechanical analysis of static forces in the thumb during hand function. J Bone Joint Surg Am.

[2] Armstrong AL, Hunter JB, Davis TR (1994). The prevalence of degenerative arthritis of the base of the thumb in post-menopausal women. J Hand Surg Br.

[3] Lane LB, Eaton RG (1987). Ligament reconstruction for the painful “prearthritic” thumb carpometacarpal joint. Clin Orthop Relat Res.

[4] Becker SJ, Briet JP, Hageman MG, Ring D (2013). Death, taxes, and trapeziometacarpal arthrosis. Clin Orthop Relat Res.

[5] Burton RI, Pellegrini VD (1986). Surgical management of basal joint arthritis of the thumb. Part II. Ligament reconstruction with tendon interposition arthroplasty. J Hand Surg Am.

[6] Pelligrini VD (1991). Osteoarthritis of the trapeziometacarpal joint: the pathophysiology of articular cartilage degeneration. II. Articular wear patterns in the osteoarthritic joint. J Hand Surg Am.

[7] Eaton RG, Littler JW (1973). Ligament reconstruction for the painful thumb carpometacarpal joint. J Bone Joint Surg Am.

[8] Kennedy CD, Manske MC, Huang JI (2016). Classifications in Brief: The Eaton-Littler Classification of Thumb Carpometacarpal Joint Arthrosis. Clin Orthop Relat Res.

[9] Epping W, Noack G (1983). Die operative Behandlung der Sattelgelenksarthrose. Handchir Mikrochir Plast Chir.

[10] Sigfusson R, Lundborg G (1991). Abductor pollicis longus tendon arthroplasty for treatment of arthrosis in the first carpometacarpal joint. Scand J Plast Reconstr Surg Hand Surg.

[11] Hays RD, Sherbourne CD, Mazel RM (1993). The RAND 36-Item Health Survey 1.0. Health Econ.

[12] Tarlov AR, Ware JE, Greenfield S, Nelson EC, Perrin E, Zubkoff M (1989). The Medical Outcomes Study. An application of methods for monitoring the results of medical care. JAMA.

[13] Hudak PL, Amadio PC, Bombardier C (1996). Development of an upper extremity outcome measure: the DASH (disabilities of the arm, shoulder and hand) [corrected]. The Upper Extremity Collaborative Group (UECG). Am J Ind Med.

[14] Michon M, Maheu E, Berenbaum F (2011). Assessing health-related quality of life in hand osteoarthritis: a literature review. Ann Rheum Dis.

[15] Faiz R, Akram RB, Hashmi FR (2025). Functional and Patient-Reported Outcomes of RegJoint™ Interpositional Arthroplasty for Thumb Carpometacarpal Joint (CMCJ) Arthritis: A Two-to-Five-Year Follow-Up Study. Cureus.

[16] Florek J, Georgiew F, Szklany K, Kotela I (2020). Evaluating outcomes of surgical treatment of patients with degenerative changes in the carpometacarpal joint of the thumb using percutaneous stabilization with Herbert screws and Reg-Joint implants - a pilot study. Chir Narzadow Ruchu Ortop Pol.

[17] Mattila S, Ainola M, Waris E (2018). Bioabsorbable poly-L/D-lactide (96/4) scaffold arthroplasty (RegJoint™) for trapeziometacarpal osteoarthritis: a 3-year follow-up study. J Hand Surg Eur Vol.

[18] Mattila S, Haapamäki V, Waris E (2020). Unfavourable early outcomes of total trapeziectomy with RegJoint™ interposition: a report of 38 osteoarthritic hands in 34 patients. J Hand Surg Eur Vol.

[19] Kennedy AM, Barker J, Estfan R, Packer GJ (2020). The use of the RegJoint™ implant for base of thumb osteoarthritis: Results with a minimum follow-up of 2 years. Hand Surg Rehabil.

[20] Erne HC, Schmauß D, Cerny M, Schmauss V, Ehrl D, Löw S, Deiler S (2017). Resektionsarthroplastik nach Lundborg vs. Pyrocarbon-Spacer (Pyrocardan®) bei fortgeschrittener Rhizarthrose – Eine Zwei-Center-Studie. Handchir Mikrochir Plast Chir.

[21] Erne HC, Cerny MK, Ehrl D, Bauer AT, Schmauss V, Moog P, Broer PN, Loew S, Schmauss D (2018). Autologous Fat Injection versus Lundborg Resection Arthroplasty for the Treatment of Trapeziometacarpal Joint Osteoarthritis. Plast Reconstr Surg.

[22] Tiihonen R, Hackenberg T, Waris E (2026). Poly-l/d-Lactide Implant (RegJoint) Versus Tendon Interposition for Trapeziometacarpal Inflammatory Arthritis: A 15-Year Follow-up of a Randomized Controlled Clinical Trial. Hand (N Y).

[23] Dietrich AKIM, Winkelmann M, O’Loughlin PF, Krettek C, Gaulke R (2022). Arthroplasty of the trapeziometacarpal joint with or without bioabsorbable polylactide scaffold (RegJoint™) interposition. Hand Surg Rehabil.

[24] Bogaert S, Cuylits N, Drossos K, Lejeune A, Tooulou M, Chahidi N (2023). Primary trapeziectomy with Regjoint(TM), a poly-L/D-lactide spacer, a two-year follow-up study with new radiological assessment tool. Acta Orthop Belg.

[25] Frey PE, Daeschler SC, Naseri Y, Franzen M, Sommer J, Harhaus L, Panzram B (2025). TOUCH® duo-mobile prosthesis in TMC osteoarthritis: two-year results and practical insights regarding key surgical steps and complication management. Arch Orthop Trauma Surg.

[26] Msallem B, Veronesi L, Halbeisen FS, Beyer M, Dragu A, Thieringer FM (2025). Evaluation of angulation and distance deviation for robot-guided laser osteotomy - a follow-up study on digital high-tech procedures. Front Robot AI.

